# The combined impact of ability, motivation, and opportunity factors on employees’ sustained workplace proactivity

**DOI:** 10.3389/fpsyg.2026.1772119

**Published:** 2026-03-11

**Authors:** Zhou Lan, Xinyue Wu, Xianghui Xing, Yunhao Wang

**Affiliations:** 1 School of Law, University of Santo Tomas, Manila, Philippines; 2 School of Law and Public Administration, Nanjing University of Information Science and Technology, Nanjing, Jiangsu, China; 3 Institute of Big Data and Rule of Law, Nanjing University of Information Science and Technology, Nanjing, Jiangsu, China

**Keywords:** AMO framework, employees, influencing factors, sustainable development, workplace proactivity

## Abstract

**Introduction:**

Employees’ workplace proactivity plays a critical role in organizational stability and sustainability.

**Methods:**

Drawing on data from the 2021 wave of the Chinese General Social Survey, this study investigates the determinants of employees’ workplace proactivity within the Ability-Motivation-Opportunity framework.

**Results:**

The results indicate that, among ability-related factors, age, job tenure, and participation in corporate training positively influence workplace proactivity. At the motivation level, being married, access to non-monetary benefits, perceived promotion opportunities, life satisfaction, interpersonal relationship satisfaction, satisfaction with work pressure, and an internal career locus of control all exert positive effects, whereas an external career locus of control negatively affects workplace proactivity. At the opportunity level, ethnicity, on-site work arrangements, and perceived labor protection enhance workplace proactivity, while overtime hours reduce it. Among them, the perceived labor protection and internal career locus of control are two core variables driving workplace proactivity. Overall, the explanatory power of motivation dimension and the opportunity dimension substantially outweighs that of the ability dimension, challenging the conventional assumption in the AMO framework of relatively balanced contributions across its three dimensions. Heterogeneity analyses show that early adulthood (age 30–39) is the stage in which the influencing factors converge most intensively; moreover, interpersonal relationship satisfaction, internal career locus of control, and perceived labor protection function as stable, cross-age drivers. A series of robustness checks confirms the stability of the findings.

**Discussion:**

This study provides policy implications for organizations seeking to enhance employee vitality and reduce turnover risk through targeted interventions in ability development, motivational enhancement, and opportunity provision. The results offer both theoretical and practical value for sustainable human resource management in the post-pandemic era.

## 1 Introduction

Currently, the global business environment faces persistent challenges such as economic uncertainty, sustained inflation, geopolitical tensions, and uneven regional development continue to shape organizational environments (United Nations, 2025). Global levels of employee workplace proactivity, measured through indicators of engagement and enthusiasm, have declined to 21%, marking the second downturn in the past decade (Gallup, 2025). Against this backdrop, organizational resilience and employee workplace proactivity have emerged as focal points in both academic research and management practice. Existing studies have confirmed that employee workplace proactivity is significantly positively correlated with organizational efficiency, productivity, and employee retention rates, serving as a core element for organizations to achieve sustainable development (OECD, 2016). With the development of digital technologies and the transformation of work models in the post-pandemic era, employees’ demands for remote or hybrid work, their emphasis on mental health, and their pursuit of work-life balance have significantly increased. These new features continue to alter and reshape the operational logic of the traditional Ability-Motivation-Opportunity (AMO) framework. Specifically, remote work environments require employees to possess higher levels of self-management and technical application abilities, prompting organizations to place greater emphasis on digital skills and psychological resilience cultivation within the ability development. Non-monetary benefits have become crucial, as employees no longer rely solely on traditional monetary incentives when choosing jobs, but instead pay more attention to their own job satisfaction and quality of life (Sharari et al., 2025). Moreover, organizations need to strengthen their focus on labor protection for employees, providing robust remote work support and flexible work systems to stimulate employees’ proactivity (Lee et al., 2025). These changes impose higher requirements on the systematic and multi-level nature of related research.

To address the global common issue of declining workplace proactivity, various countries have enacted relevant policies focusing on employee rights protection and optimization of the work environment, which align closely with the analytical framework of this study. The United States has alleviated employees’ work pressure through policies such as mental health benefits and flexible work arrangements, emphasizing incentives and emotional guidance at the motivation dimension. Singapore has introduced guidelines on flexible work arrangements, satisfying employees’ personalized demands for work location and timing from the opportunity dimension (Shanghai Intelligence Service Platform, 2024). Canada, meanwhile, strengthens organizational support and rights protection at the opportunity dimension through regulations on pay equity and labor rights safeguards (McMillan, 2023). These international practices offer directional insights for elevating workplace proactivity and highlight the significance of ability cultivation, motivation stimulation, and opportunity provision.

Stimulating employee workplace proactivity, enhancing employee engagement, and constructing an efficient and sustainable labor force represent the core proposition for corporates in addressing market competition and realizing high-quality development. This is also a critical pathway to advancing improvements in employment quality and ensuring the sustainable functioning of the labor market, and represents an important agenda of widespread concern across sectors in the current context of digital transformation and the rise of new forms of employment (Hanu and Khumalo, 2024). At present, research integrating the AMO framework into strategic human resource management in Chinese organizations remains insufficient, making it difficult to develop intervention strategies tailored to the characteristics of local employees. Against the backdrop of post-pandemic workplace features and the contemporary era, systematically investigating the influencing factors and underlying mechanisms of employee workplace proactivity in the Chinese context during the post-pandemic era holds significant theoretical value and practical significance.

The AMO framework offers a fitting analytical lens for the research outlined above. Its core advantage lies in systematically analyzing the three mechanisms of ability empowerment, motivation activation, and opportunity provision, thereby achieving a multi-level integration of the influencing factors on employee workplace proactivity. This matches the complex formation logic of workplace proactivity as an interplay of “individual intrinsic ability – subjective motivation – external environmental support.” Meanwhile, the open-ended design of the “opportunity” dimension effectively integrates emerging contextual variables in the post-pandemic era, such as work arrangements and perceived labor protection, meeting new research demands and making it an appropriate method for analyzing determinants of Chinese employees’ workplace proactivity. The 2021 data from the Chinese General Social Survey (CGSS) provides critical support for the empirical analysis of this study. The CGSS can reflect the overall characteristics of employees across different regions and industries in China. The dataset includes indicators corresponding to the ability dimension (e.g., demographic, job tenure, corporate training), motivation dimension (e.g., marital status, life satisfaction, occupational beliefs), and opportunity dimension (e.g., work arrangement preferences, perceived labor protection, overtime hours). These variables align closely with the empirical requirements of the AMO framework, effectively overcoming the deficiencies prevalent in prior studies – such as inadequate empirical evidence in the Chinese context, unidimensional variable selection, and limited sample representativeness. The dataset also supports subsequent heterogeneity and robustness tests, providing a reliable data foundation for accurately identifying the drivers of workplace proactivity among Chinese employees.

In summary, this study takes the 2021 wave of CGSS data as its sample and systematically investigates the influencing factors of employee workplace proactivity in China based on the AMO framework. The main research questions include: (1) Within the three dimensions of the AMO framework-ability, motivation, and opportunity-what specific factors influence employee workplace proactivity? (2) What are the directional effects and underlying mechanisms of these dimensional factors on Chinese employee workplace proactivity? (3) Based on empirical findings and international practical experience, how can Chinese corporates and relevant departments implement targeted interventions across the ability, motivation, and opportunity dimensions to enhance employee workplace proactivity? The contributions of this study are as follows: (1) At the theoretical level, first, grounded in the post-pandemic workplace realities in China, it deepens the localization and contextual adaptation of the AMO framework. By overcoming the single-dimensional focus of prior research, it systematically elucidates the synergistic mechanisms and reshaping logic across the ability, motivation, and opportunity dimensions, thereby refining the theoretical explanatory system for the formation of employee workplace proactivity in the new era. Second, the study incorporates emerging realities overlooked in previous work—such as the widespread adoption of remote work, policy support for new employment forms, and accelerating population aging—to analyze the influencing factors of workplace proactivity within the AMO framework. This approach addresses the disconnect between existing theoretical conclusions and employees’ current work scenarios and needs, while expanding the framework’s applicability in diverse contemporary workplace contexts. (2) At the practical level, first, based on empirical data from the Chinese context, this study identifies the core drivers of workplace proactivity and its age-based heterogeneity, thereby providing an evidence-based foundation for corporates to design tiered and targeted intervention programs to enhance workplace proactivity. Second, by integrating international policy orientations, it offers valuable references for China to improve its labor rights protection system and optimize human resource management policies, ultimately supporting the realization of sustainable human resource management in Chinese corporates during the post-pandemic era.

In addition to the AMO framework, a variety of complementary theories provide diverse perspectives and theoretical support for understanding the formation mechanisms of employee workplace proactivity. Van den Broeck et al. (2021) extended Self-Determination Theory (SDT), highlighting that autonomy, competence, and relatedness are key elements in sustaining employees’ ongoing work engagement. The Job Demands-Resources model (JD-R), from the perspective of work-context interaction, elucidates the critical value of resource provision in alleviating demand pressure, preventing employee burnout, and enhancing workplace proactivity (Huang et al., 2025). The Conservation of Resources theory (COR) further reveals the psychological logic underlying employees’ resource acquisition and conservation, offering a theoretical foundation for understanding the linkage between organizational resource support and employee workplace proactivity. Social Exchange Theory, grounded in the principle of reciprocity, explains the positive shaping effect of the exchange relationship between organizations and employees on employee attitudes and behaviors (Smith, 2002). This study deeply integrates the AMO framework with SDT, JD-R, COR, and Social Exchange Theory, taking the workplace realities of post-pandemic China as the contextual backdrop. By embedding the core constructs and analytical perspectives of these theories into the ability, motivation, and opportunity dimensions of the AMO framework, this study builds a more systematically integrated and contextually grounded research framework for examining employee workplace proactivity.

## 2 Literature review

A review of existing scholarship reveals a clear developmental trajectory in research on employee workplace proactivity — from the initial conceptualization and the development of measurement instruments to systematic examinations of influencing factors, and more recently, to the integration of theoretical frameworks and in-depth analyses of underlying mechanisms.

The 1990s marked the nascent stage of research on workplace proactivity, during which scholars primarily focused on clarifying the construct and developing measurement tools (Ribisl and Reischl, 1993; Herrenkohl et al., 1999). In fact, as work environments have continued to evolve and scholarly inquiry has deepened, these measurement instruments have undergone continual refinement. For instance, Mbindyo et al. (2009) developed a tool grounded in seven structural dimensions to assess motivation among medical personnel in Kenyan hospitals. Pancasila et al. (2020) designed a scale to investigate how workplace proactivity and leadership shape employee job satisfaction and performance. Abdullah et al. (2025) integrated the five objectives of Maqasid Shariah with McClelland’s need theory and proposed 15 propositions to explain and measure employee positivity.

Entering the 21st century, research on employee workplace proactivity has begun to develop in greater depth, with scholars examining multiple influencing factors from diverse theoretical perspectives. Commonly studied determinants include leadership styles, green human resource management (Shahzad et al., 2025), organizational culture (Jo and Shin, 2025), and so on. Leadership style, as a core element of organizational management, exerts a profound influence on employee workplace proactivity. A representative example is Mphaluwa et al. (2025), who employed a cross-sectional survey design to examine the effects of transformational, democratic, and authoritarian leadership styles on employee performance. Simultaneously, the organizational environment, as the immediate context of employees’ work, systematically affects workplace proactivity. Environmental factors include organizational support (Niraula et al., 2025), compensation systems (Igalens and Roussel, 1999), and organizational culture (Paais and Pattiruhu, 2020), among other dimensions, which influence employees’ attitudes and behaviors through various psychological mechanisms. For instance, Jawahar and Stone (2011) incorporated perceptions of pay fairness and satisfaction into their analytical framework, reflecting a multifactor interactive research approach. Third, employees’ individual characteristics, as intrinsic determinants of workplace proactivity, have attracted wide research attention. These factors include psychological capital (Zhang and Bartol, 2010), occupational identity (Canrinus et al., 2012), and demographic characteristics (Kukanja, 2013), which affect employees’ work engagement through internal motivational processes. Fourth, employees in different occupational groups exhibit significant variation in both determinants and manifestations of workplace proactivity due to differences in job nature, occupational environment, and social expectations. Existing studies have also conducted targeted investigations of workplace proactivity in specific fields, such as education (Layek and Koodamara, 2024), healthcare (Kyambade and Namatovu, 2025), and the public sector (Wightman and Christensen, 2025). Such research on particular occupational groups contributes to understanding the contextual specificity of workplace proactivity.

In recent years, research on employee workplace proactivity has deepened, with theoretical development continuing and methodological approaches becoming increasingly diverse. For example, Hajiali et al. (2022) employed AMOS 18 and structural equation modeling to analyze the effects of workplace proactivity, leadership style, and employee ability on job satisfaction. Similarly, Park et al. (2024), using survey data on career development pathways, demonstrated the significant effectiveness of machine learning in predicting turnover intention, with its performance surpassing that of traditional econometric models. Furthermore, Qin et al. (2025) integrated artificial intelligence into employee-related research, finding that employee AI identity significantly affects proactive work behavior, with proactive work intention serving as a mediating factor.

Existing research in the domain of employee workplace proactivity has accumulated a certain body of findings, providing a more solid foundation for comprehensively analyzing the influencing mechanisms and practical interventions related to workplace proactivity. However, significant research gaps remain. First, the research perspective remains relatively narrow. Most existing studies focus predominantly on explicit incentive factors—such as compensation and benefits (Segura-Camacho et al., 2018; De Jonge et al., 2000; Rynes et al., 2004), promotion channels (Setyawati et al., 2022; Haryono et al., 2020), and managerial characteristics (Webb, 2007; Campos-García and Zúñiga-Vicente, 2019)—or macro-level organizational institutional arrangements. There has been insufficient in-depth exploration of mechanisms involving employees’ individual characteristics, situational perceptions, and emotional responses, making it difficult to fully reveal the diverse formation pathways of workplace proactivity. Second, multi-level integrative research requires further strengthening. The majority of studies remain confined to a single level of analysis and lack systematic integration across multiple dimensions—including individual, organizational, and environmental factors—thereby failing to align with the complex formation logic of workplace proactivity. Third, constrained by data timeliness and limited research perspectives, prior studies have not adequately captured the evolving characteristics of workplace proactivity under new realities, such as the widespread adoption of remote work, improved policy support for emerging employment forms, and accelerating population aging. This has resulted in a disconnect between certain theoretical conclusions and the realities of today’s work situations and employee needs. These research gaps offer important opportunities for further systematic examination of the influencing factors, heterogeneity characteristics, and deeper underlying mechanisms of employee workplace proactivity.

## 3 Theoretical analysis and research hypotheses

The AMO framework (Appelbaum et al., 2001; Boxall and Purcell, 2003) has been widely accepted as a central theoretical lens for explaining the linkage between human resource management (HRM) practices and organizational performance. The AMO theory posits that HRM influences organizational performance through three mechanisms: (1) Ability: enhancing employees’ knowledge, skills, and other competencies through education and training; (2) Motivation: stimulating employees’ willingness to perform tasks through mechanisms such as recognition, performance-based pay, and incentive compensation; (3) Opportunity: improving the external work environment through resource provision, leadership behaviors, work practices, and organizational strategies that create opportunities for high employee involvement in HRM.

### 3.1 Ability (A)

Ability refers to the physiological and cognitive capabilities that enable an individual to perform tasks effectively (Blumberg and Pringle, 1982) or more broadly, employees’ knowledge, skills, competencies, and proficiencies (Kim et al., 2015; Marin-Garcia and Martinez Tomas, 2016). These capabilities can be strengthened through recruitment, training, and development initiatives, enabling employees to carry out workplace tasks effectively. Employees’ ability levels directly influence both their subjective attitudes and objective performance. The accumulation of knowledge, skills, and experience can enhance employees’ confidence and sense of efficacy, thereby promoting greater workplace proactivity. Importantly, ability is not merely an innate endowment but a dynamic factor that can be improved through cultivation and accumulation over time. HRM theory suggests that employees with stronger abilities are more likely to exhibit higher levels of work engagement and positive affect because they are better equipped to handle job challenges and achieve self-efficacy.

Age, job tenure, and active participation in organizational training can be regarded as proxy indicators of accumulated ability. These factors positively influence employee workplace proactivity by enhancing experience accumulation and skill acquisition. Age is associated with individual maturity and accumulated experience. As individuals grow older, they typically acquire richer life and work experience that helps them develop more stable and positive workplace attitudes. Prior studies show that older employees tend to seek intrinsically challenging work (Boumans et al., 2011) and often display higher job satisfaction and positivity due to stronger problem-solving skills and better emotional regulation capabilities (Kalleberg and Loscocco, 1983). Similarly, job tenure reflects the duration of an employee’s involvement in a specific field or organization. Through continuous practice and learning, employees enhance their capabilities, which in turn increases their enthusiasm and engagement at work. Experienced employees are generally more effective at handling complex tasks, reducing frustration, and translating competence into higher workplace proactivity (Locke and Latham, 1990). Furthermore, participation in corporate training is a direct channel for ability enhancement. Through systematic knowledge transfer and skill training, employees not only strengthen job competencies but also stimulate intrinsic motivation and positive emotions through enhanced professional capabilities. Based on the above analysis, we propose Hypothesis 1.

Hypothesis 1: Age, job tenure, and participation in corporate training have a positive impact on workplace proactivity.

### 3.2 Motivation (M)

Motivation can be understood as the force that directs, energizes, and sustains behavior (Van Iddekinge et al., 2018) or as employees’ willingness and desire to perform a task. Internal and external factors such as rewards, satisfaction, beliefs, and expectations jointly drive employee workplace proactivity and persistence. Employees with stronger motivation tend to demonstrate higher levels of work engagement and more positive attitudes because they perceive value and returns from their work and are better able to align personal goals with organizational objectives. Prior research commonly distinguishes between intrinsic motivation and extrinsic motivation. Intrinsic motivation derives from sources such as a sense of achievement and personal beliefs (Froman, 2010), whereas extrinsic motivation stems from benefits (Sackey et al., 2025) and promotion opportunities (Liu et al., 2024). Both types positively influence workplace behavior by satisfying employees’ psychological and social needs. When individuals perceive that their needs are met, that effort is linked to rewards, and that career development is predictable, workplace proactivity is substantially activated. Conversely, negative psychological cognitions and beliefs may suppress employees’ willingness to engage proactively.

Marital status, non-monetary benefits, perceived opportunities for future promotion, life satisfaction, interpersonal relationship satisfaction, satisfaction with work pressure, internal career locus of control, and external career locus of control all influence employees’ workplace proactivity by enhancing intrinsic fulfillment or strengthening external incentives.

Marital status shapes individuals’ living conditions and sense of responsibility, thereby affecting workplace proactivity. Married employees typically face clearer family responsibilities and develop stronger preferences for job stability and career advancement, leading to more goal-oriented work engagement. Additionally, marital status is closely associated with life stability and support networks. Married individuals often receive emotional support from family members (Demerouti et al., 2012), which helps mitigate work-related stress and sustain a positive work mindset.

Non-monetary benefits, particularly health protection schemes, paid leave policies, and additional non-cash incentives, represent organizational investments beyond financial compensation and reflect organizational attention to employees’ personal needs. Satisfying these non-material needs enhances job satisfaction and motivation level because employees interpret such benefits as signals of organizational care, and they are likely to reciprocate with greater work engagement (Waqas and Saleem, 2014). Moreover, non-monetary benefits can effectively alleviate life and work pressures, reduce concerns outside the workplace, and create favorable conditions for proactive work involvement.

Perceived opportunities for future promotion reflect employees’ career expectations, namely their subjective assessment of career development prospects. When employees perceive that their efforts can lead to substantive returns such as promotion opportunities, their sense of purpose at work and motivational intensity are significantly enhanced. The career advancement space associated with promotion also satisfies individuals’ needs for achievement and self-actualization, thereby continuously stimulating workplace proactivity.

Life satisfaction represents individuals’ overall evaluation of their living conditions and reflects their broader sense of well-being. High life satisfaction indicates that employees’ needs in non-work domains, such as family and social life, are adequately met, enabling them to devote greater energy and a more positive mindset to work. A positive life state also enhances individuals’ emotional regulation capacity, reducing the disruptive influence of negative emotions on work (Mafini and Dlodlo, 2014) and, in turn, promoting workplace proactivity. Interpersonal relationship satisfaction concerns the quality of employees’ interactions with colleagues. A constructive workplace relationship network fosters a harmonious work environment, reduces interpersonal friction and the stress costs associated with conflict, while also providing emotional support and collaborative resources in work settings (Yu et al., 2007). Such high-quality interactions strengthen employees’ sense of belonging and recognition. Satisfaction with work pressure reflects the extent to which employees accept the current intensity of work demands, focusing on the perceived match between pressure levels and personal coping capabilities. When work pressure remains within an acceptable and manageable range, it can stimulate employees’ desire for challenge and allow them to gain a sense of achievement during task completion (Jamal, 1990). Conversely, excessive work pressure may lead to overload and occupational burnout.

An internal career locus of control refers to individuals’ belief that their career development and work outcomes are primarily determined by internal factors such as effort and capability. This belief strengthens employees’ sense of agency and responsibility toward career achievements, motivating them to set proactive goals, respond actively to challenges, and attribute work outcomes to personal effort, thereby sustaining their willingness to engage proactively. It also enhances resilience when difficulties arise and reduces the negative influence of external fluctuations. In contrast, an external career locus of control reflects the belief that career development and work outcomes depend on external forces such as luck, environment, or the decisions of others. This belief fosters workplace passivity, weakens self-efficacy, and reduces employees’ willingness to exert control over their work. Individuals with a stronger external orientation tend to attribute success or failure to uncontrollable conditions, thereby diminishing the motivation to actively engage. Moreover, they are more likely to feel helpless when encountering work pressure or setbacks, which undermines workplace proactivity. Based on the above analysis, we propose Hypothesis 2:

Hypothesis 2: At the motivation level, being married, non-monetary benefits, perceived opportunities for future promotion, life satisfaction, interpersonal relationship satisfaction, satisfaction with work pressure, and an internal career locus of control positively affect workplace proactivity, whereas an external career locus of control negatively affects workplace proactivity.

### 3.3 Opportunity (O)

Opportunity encompasses situational or environmental factors beyond individuals’ direct control, generally referring to the contextual and structural support provided by organizations (Gartner et al., 2008), such as job design, resources, and participation opportunities that enable employees to apply their abilities and motivation. Opportunity has been described by Blumberg and Pringle (1982) as “the field of forces surrounding a person and his or her tasks that enables or constrains that person’s task performance.” Within HRM theory, opportunity is viewed as a contextual factor that amplifies the effects of individual ability and motivation. When opportunities are lacking, employees may be unable to demonstrate optimal performance even when they possess strong abilities and high motivation.

Ethnicity, work arrangement preferences, perceived labor protection, and overtime intensity may influence employee workplace proactivity through mechanisms related to organizational inclusiveness, work flexibility, and workload management.

Ethnicity reflects individuals’ or groups’ cultural identities. Differences in professional values, teamwork preferences, and perceptions of organizational belonging may exist across ethnic groups. Many countries have enacted employment-support and cultural-protection policies targeting minority or specific ethnic groups, alongside corresponding multicultural inclusion mechanisms within workplaces. These measures provide institutional safeguards for employees of diverse ethnic backgrounds to integrate into their work environments. In multicultural workplace environments, national-level ethnic policies align with organizational cultural inclusion systems, thus respecting the cultural customs and developmental needs of ethnic minorities while reinforcing group identity and cultural compatibility through institutional design. Consequently, it influences employees’ acceptance of the work environment and their willingness to commit. Research indicates that when organizations effectively implement ethnicity-related policy requirements and formally ensure equal rights and cultural inclusion for all ethnic groups through institutional safeguards, employees are more likely to develop emotional attachment and institutional trust toward the workplace (Amah and Oyetunde, 2019).

Work arrangement preference reflects employees’ individualized needs regarding work settings and time structures. Remote work has emerged as a central workplace demand in the post-pandemic era, and the advancement of digital technologies has further enhanced its feasibility. Some scholars argued that the contemporary workplace has entered a new era in which alternative work arrangements, particularly remote work, have become normalized (Gajendran et al., 2024; Leonardi et al., 2024). Preferences for remote, on-site, or hybrid work may shape workplace proactivity through different motivational pathways. From the perspective of SDT, on-site work provides immediate guidance and collaborative support that help employees acquire work skills more efficiently, enhance task performance, and strengthen perceived competence. Physical workspaces also fulfill social needs and reduce isolation by providing interactive scenarios, while flexible in-person communication improves employees’ sense of autonomy and control over work. When individuals’ psychological needs for autonomy, competence, and relatedness are met, their intrinsic motivation is significantly heightened (Ryan and Deci, 2000). Moreover, recent studies have found that remote and hybrid work models reduce the quality and frequency of innovative ideas, underscoring the challenges organizations face in sustaining innovation without regular in-person interaction (Dwivedi et al., 2023).

Perceived labor protection reflects employees’ subjective assessment of the extent to which their legitimate rights are safeguarded, directly signaling organizational responsibility. Eisenberger et al. (2001) suggested that employees in an organization can exchange commitment in the reciprocation of organizational support. Individual behavior in the workplace essentially follows the logic of reciprocity: the quality of employee-organization interactions directly influences attitudes and behavioral outcomes. When employees perceive that their legitimate labor rights are valued and protected, they reciprocate through higher work engagement. Conversely, insufficient labor protection leads to dissatisfaction and diminished workplace proactivity.

Overtime days serve as a key indicator of workload. Excessive overtime reduces rest and leisure time, disrupts work-life balance, and contributes to physical and emotional fatigue and occupational burnout. Long-term heavy overtime may also deteriorate employees’ health conditions (Dembe et al., 2005). According to the COR, individuals’ time and energy are finite; chronic overtime depletes resources continuously with insufficient replenishment, ultimately reducing work enthusiasm and proactive engagement and exerting a negative influence on workplace proactivity. Based on the above analysis, we propose Hypothesis 3:

Hypothesis 3: At the opportunity level, ethnicity, on-site work arrangements, and perceived labor protection positively influence workplace proactivity, whereas overtime days negatively influence workplace proactivity.

## 4 Data selection and research design

### 4.1 Sample selection and data sources

This study uses data from the 2021 wave of the CGSS, which is accessed from the data archive^1^. Initiated in 2003, CGSS is the earliest nationwide, comprehensive, and continuous academic survey project in China. It systematically collects data at multiple levels, including society, community, family, and individuals, summarizes trends of social change, explores issues with significant scientific and practical implications, promotes openness and data sharing in domestic research, and provides data materials for international comparative studies. It also functions as a multidisciplinary platform for the collection of economic and social statistics. However, the CGSS 2021 enterprise employee special survey used in this paper is limited to five provinces—Beijing, Shanghai, Guangzhou, Sichuan, and Shaanxi. It employed a multi-stage stratified probability sampling method to interview employees from 250 companies, ultimately yielding a sample size of 3,781 observations. This study complies with ethical standards and has been approved by the Ethics Committee of the School of Law and Public Administration at Nanjing University of Information Science and Technology. Written informed consent was obtained from all participants. This statement clarifies that the original data collection process was managed by the CGSS administrators, who obtained informed consent from participants at the time of data collection. Consequently, no additional consent from participants is required when conducting secondary analysis using these de-identified data. Furthermore, the Ethics Committee of the institution conducting this research has reviewed the relevant secondary de-identified analysis and confirmed its compliance with ethical standards. Under applicable regulations, de-identified data analysis does not necessitate further participant consent, and such studies are considered exempt. Specifically, the dataset used in this study was fully anonymized by the data provider prior to public release to ensure the protection of individual privacy. All procedures adhered to the ethical standards outlined in the Declaration of Helsinki.

As the CGSS 2021 survey data do not include sampling weights, this study refrains from weighted estimation. All statistical analyses are conducted based on the original unweighted sample. After defining key variables, the data were processed and analyzed using Stata 18. The sample is strictly restricted to respondents who provided valid answers on all key variables, and the following screening criteria were applied to ensure sample validity:

1. Excluding labor-dispatched workers.
2. Excluding interns.
3. Excluding questionnaires mistakenly filled in using the 2020 version.

After applying these filters, a total of 3,287 valid observations were obtained.

### 4.2 Variable definitions

#### 4.2.1 Dependent variable

The dependent variable in this study is organizational commitment, which is defined as employees’ emotional attachment to, identification with, and desire to maintain long-term membership in the organization (Meyer and Allen, 1991). The scale consists of four items: “1. I have an emotional attachment to this organization; 2. I treat the organization’s problems as my own problems; 3. I hope to work in this organization for a long time; 4. I care deeply about the organization’s future and destiny.” The scale measures employees’ positive attitudes and behavioral inclinations toward the organization, including emotional identification (Item 1), internalized sense of responsibility (Item 2), continuance commitment (Item 3), and value integration (Item 4). These components align closely with employees’ psychological ownership, proactive work behaviors, and willingness to remain bound to the organization in the workplace. Each item is measured on a 0–7 scoring scale. After assigning equal weights to the four items, their scores are summed to form a composite index of organizational commitment. Existing research indicates that organizational commitment serves as a key psychological foundation and proximal indicator for employees’ display of proactive workplace behaviors—such as extra-role behavior (Davoudi, 2012), proactive behavior (Crant, 2000), innovative behavior (Hakimian et al., 2016; Jafri, 2010), and others. Therefore, this study selects organizational commitment as the dependent variable to quantify employees’ positive workplace tendencies. Specifically, the higher the composite score of organizational commitment, the greater the corresponding level of workplace proactivity.

#### 4.2.2 Independent variables

The independent variables fall into four categories: demographic characteristics, economic and work characteristics, psychological and attitudinal characteristics, and social security and labor-rights characteristics. Demographic characteristics include age, gender, ethnicity, marital status. Economic and work characteristics are primarily examined from three dimensions: work income and benefits, workload and work arrangement, and career development. Specifically, these variables include average monthly income, annual bonus, non-monetary benefits, average weekly overtime (in days), work arrangement preference, job tenure, technical or positional promotion experiences, and perceived opportunities for future promotion. Psychological and attitudinal characteristics capture emotional status, satisfaction, and personality traits and tendencies. These include satisfaction with living conditions, interpersonal relationships, and work pressure, as well as internal career locus of control and external career locus of control. Social security and labor-rights characteristics focus on insurance coverage and labor-rights management, including participation in corporate annuity programs, labor contract signing status, perceived labor protection, and participation in corporate training programs. Table 1 presents the definitions and measurement methods of all key variables used in this study.

**Table 1:** Definitions and measurement of key variables.

Variable type	Variable name			Symbol	Definition and measurement
Dependent variable	Organizational commitment			*WorkMot*	1. I have an emotional attachment to this organization. 2. I treat the organization’s problems as my own problems. 3. I hope to work in this organization for a long time. 4. I care deeply about the organization’s future and destiny. These items use a 0–7 scale, ranging from “strongly disagree” to “strongly agree.” Equal weights (1:1:1:1) are assigned, and the sum is used as the composite measure.
Independent variable	Demographic characteristics	Age		*Age*	The respondent’s actual age at the time of the 2021 interview.
		Gender		*Gender*	Male = 1; Female = 0.
		Ethnicity		*National*	Han = 0; non-Han = 1.
		Marital Status		*Marry*	Unmarried, Widowed, Divorced and Not Remarried = 0; Married (first marriage or remarriage) = 1.
	Economic and work characteristics	Work income and benefits	Average monthly income	*MonNetInc*	Logarithm of average monthly after-tax income of previous year, specifically using the formula log (x + 1), where x is the original value.
			Annual bonus	*AnnBonus20*	Logarithm of annual bonus in the previous year, specifically using the formula log (x + 1), where x is the original value.
			Non-monetary benefits	*Welfare*	Number of non-monetary benefits provided by the corporate, including free meals, free or low-cost housing, free access to sports and cultural facilities, full-time training during employment, employer-sponsored study programs, paid leave, regular cultural activities, internal promotion, and company-organized trips.
		Workload and work arrangement	Average weekly overtime (in days)	*OTDaysPWLM*	Average number of overtime days per week in the month preceding the interview date.
			Work arrangement preference	*WorkFormPref*	Respondents’ preferred work arrangements were categorized and coded as follows: Remote = 1; Hybrid = 2; On-site = 3.
			Job tenure	*CurrTenure*	The number of years the respondent has worked at the current organization as of the interview date.
		Career development	Technical or positional promotion experiences	*Promote3yr*	Technical or positional promotion experience in the past 3 years: Promoted = 1; Not Promoted = 0.
			Perceived opportunities for future promotion	*PromoteFut*	Subjective expectation of promotion in the next few years: Almost Impossible = 1; Unlikely = 2; Uncertain/Difficult to Judge = 3; Likely = 4; Almost Certain = 5.
	Psychological and attitudinal characteristics	Emotional status and satisfaction	Life satisfaction	*LifeSat*	Life satisfaction degree: Very Dissatisfied = 1; Dissatisfied = 2; Neutral = 3; Satisfied = 4; Very Satisfied = 5.
			Interpersonal relationships satisfaction	*RelatSat*	Interpersonal relationship satisfaction degree: Very Dissatisfied = 1; Dissatisfied = 2; Neutral = 3; Satisfied = 4; Very Satisfied = 5.
			Work pressure satisfaction	*WorkPresSat*	Satisfaction with work pressure one received: Very Dissatisfied = 1; Dissatisfied = 2; Neutral = 3; Satisfied = 4; Very Satisfied = 5.
		Personality traits and tendencies	Internal career locus of control	*IntCareBel*	1. Job opportunities are created by oneself. 2. Anyone can achieve the work they want to accomplish whatever they do. 3. What one does now determines one’s future. 4. Most people can handle their jobs as long as they work hard. Strongly Disagree = 1; Disagree = 2; Uncertain = 3; Agree = 4; Strongly Agree = 5. Equal weights (1:1:1:1) are assigned, and the sum is used as the composite measure of internal locus of control career belief.
			External career locus of control	*ExtCareBel*	1. Getting the job one wants mainly depends on luck. 2. Making money mainly depends on fortune. 3. Getting a good job must rely on help from family and friends. Strongly Disagree = 1; Disagree = 2; Uncertain = 3; Agree = 4; Strongly Agree = 5. Equal weights (1:1:1) are assigned, and the sum is used as the composite measure of external locus of control career belief.
	Social security and labor-rights characteristics	Insurance coverage	Participation in corporate annuity programs	*EAnnuity*	Corporate annuity participation (corporate supplementary pension): Participated = 1; Not Participated or Unclear = 0.
		Labor-rights management	Labor contract signing status	*SignContract*	Signed = 1; Not Signed = 0.
			Perceived labor protection	*LabProtect*	Measures the respondents’ perception of the labor protection measures at their companies. Perceived labor protection: Very poor = 1; Poor = 2; Fair/Unclear = 3; Good = 4; Very Good = 5.
			Participation in corporate training programs	*CoTraining*	Participation in company-provided training. Participated = 1; Not Participated = 0.

### 4.3 Correlation analysis

Before conducting the regression analysis, the study performed correlation tests between the dependent variable and the independent variables selected from four dimensions: demographic characteristics, economic and work characteristics, psychological and attitudinal characteristics, and social security and labor-rights characteristics. Depending on variable types, chi-square tests and Spearman correlation analyses were applied separately. The results are presented in Tables 2, 3.

**Table 2:** Chi-square test results.

Independent variable	Subrow	Χ^2^ value (df)	*P*-value
*Gender*	Male	38.959 (28)	0.082
	Female		
*National*	Han	18.576 (28)	0.911
	Non-Han		
*Marry*	Unmarried, Widowed, Divorced and Not Remarried	138.190 (28)	<0.001
	Married (first marriage or remarriage)		
*WorkFormPref*	Remote	106.317 (56)	<0.001
	Hybrid		
	On-site		
*Promote3yr*	Promoted	70.785 (28)	<0.001
	Not Promoted		
*EAnnuity*	Participated	55.128 (28)	0.002
	Not Participated		
*SignContract*	Signed	60.622 (28)	<0.001
	Not Signed		
*CoTraining*	Participated	89.856(28)	<0.001
	Not Participated		

*p*-values < 0.05 indicate statistically significant associations.

**Table 3:** Spearman correlation analysis.

Independent variable	Spearman’s *ρ*	*p*-value
*Age*	0.193	<0.001
*MonNetInc*	0.132	<0.001
*AnnBonus20*	0.115	<0.001
*Welfare*	0.196	<0.001
*CurrTenure*	0.123	<0.001
*OTDaysPWLM*	−0.025	0.145
*PromoteFut*	0.246	<0.001
*LifeSat*	0.237	<0.001
*RelatSat*	0.288	<0.001
*WorkPresSat*	0.195	<0.001
*IntCareBel*	0.339	<0.001
*ExtCareBel*	−0.095	<0.001
*LabProtect*	0.331	<0.001

Correlation coefficients range from −1 to 1; larger absolute values indicate stronger correlations.

Based on the results in Table 2, marital status, work arrangement preference, technical or positional promotion experiences, participation in corporate annuity programs, labor contract signing status, and participation in corporate training programs all show significant associations with employee organizational commitment. Variables such as corporate annuity participation, labor contract signing status, and corporate training participation reflect, to some extent, employees’ access to economic and social resources. Differences in resource access directly shape employees’ sense of career security, which in turn influences their affective commitment and long-term willingness to invest in the organization. Meanwhile, corporate annuity participation, labor contract signing, and training participation represent the organization’s formal commitments to employees; past promotion experience and work arrangement preference reflect employees’ expectations regarding career development pathways. These factors jointly contribute to employees’ labor input and long-term work intentions, ultimately manifesting as significant associations with organizational commitment.

In summary, except for gender and ethnicity, all selected variables show significant associations with organizational commitment (*p* < 0.05). These associations span personal characteristics, economic and work characteristics, and social security and labor-rights characteristics, suggesting that workplace proactivity arises from the interplay of multidimensional factors. It should be noted that the chi-square test only indicates associations among variables, not causal directions, laying groundwork for subsequent analyses.

Table 3 presents the correlations between continuous independent variables and employee organizational commitment. In terms of coefficient magnitudes, internal locus of control career belief (0.339), perceived labor protection (0.331), and interpersonal relationship satisfaction (0.288) exhibit relatively strong positive correlations with organizational commitment; most remaining variables show moderate or weak positive or negative correlations. Most *p*-values are far below 0.05, indicating high statistical significance.

Among these, the internal locus of control career belief measures individuals’ subjective belief in controllability and self-responsibility within their career domain. Its strongest positive correlation highlights employees’ perceived control over their career development. Perceived labor protection captures employees’ subjective cognition and assessments of workplace safety and health protection measures, management practices, and the effectiveness of protection mechanisms. It reflects employees’ personal experiences and trust in workplace safety and effectiveness of institutional safeguards. Such perceptions can enhance employees’ organizational identification and sense of career security by conveying organizational care, thereby influencing workplace proactivity. Meanwhile, satisfaction with life and interpersonal relationships provides stable support from the dimensions of physical and mental well-being and social environment, enabling employees to invest more energy and willingness in their work.

### 4.4 Model construction

To analyze the determinants of employee workplace proactivity, the study constructs a linear regression model and employs ordinary least squares (OLS) estimation for the regression analysis. The primary rationale for selecting this method is as follows: the data used in this study are cross-sectional, and the core dependent variable “organizational commitment” is a continuous composite index constructed by summing the items. OLS linear regression provides directly interpretable marginal effect estimates of the causal relationships between variables, aligning well with the study’s core analytical objective of examining the impact of various factors on employee workplace proactivity. At the same time, recognizing that cross-sectional data are prone to heteroskedasticity, which may bias the estimation results, all regression analyses in this study apply White’s robust standard errors to adjust the model. This adjustment enhances the reliability and robustness of the estimates, thereby ensuring the rigor and credibility of the research conclusions.

Based on the correlation results, 21 indicators were ultimately selected as independent variables, representing demographic characteristics (*Demo*), economic and work characteristics (*EconWork*), psychological and attitudinal characteristics (*PsyAtt*), and social security and labor-rights characteristics (*SocProt*). Given the large number of variables, these four abbreviations are used to denote the independent variables. *ε* represents the random error term. With “organizational commitment” as the dependent variable, the study constructs an OLS multiple linear regression model. The regression model is specified as shown in Equation 1.


$$\begin{array}{l} \text{WorkMot}=\beta_{0}+\beta_{1}\cdot \text{Demo}+\beta_{2}\cdot \text{EconWork}\\ +\beta_{3}\cdot \text{PsyAtt}+\beta_{4}\cdot \text{SocProt}+\varepsilon \end{array}$$ (1)

## 5 Empirical results analysis

### 5.1 Reliability and validity tests of the questionnaire

For reliability, Cronbach’s Alpha was used to assess the internal consistency of the four scoring items under the variable “organizational commitment.” Cronbach’s Alpha measures the degree of consistency among items, with values ranging from 0 to 1; higher values indicate better internal consistency. As shown in Table 4, the overall Cronbach’s Alpha coefficients for *WorkMot, IntCareBel, ExtCareBel,* and *WorkDisf* are 0.929, 0.783, 0.779, and 0.920, respectively*. WorkDisf* is the reverse-coded expression of organizational commitment and is used as an alternative dependent variable in the subsequent robustness test. Usually, a Cronbach’s Alpha above 0.7 is considered acceptable, above 0.8 good, and above 0.9 excellent. Thus, all scales in this study have at least acceptable internal consistency, indicating good reliability and providing a sound foundation for subsequent analyses.

**Table 4:** Reliability test results.

Dimensions	Cronbach’s **α**	Number of items
*WorkMot*	0.929	4
*IntCareBel*	0.783	4
*ExtCareBel*	0.779	3
*WorkDisf*	0.920	4
*Overall scale*	0.848	15

For validity, Bartlett’s Test of Sphericity and the Kaiser-Meyer-Olkin (KMO) measure were applied, with results shown in Table 5. Bartlett’s test shows *p*-values below 0.001 (*p* < 0.001), far below the 0.05 significance threshold, indicating significant correlations among variables and rejecting the null hypothesis of no correlation, thus supporting the appropriateness of factor analysis. KMO is used to measure the degree of commonality among variables, with values ranging from 0 to 1. Generally, KMO values ≥0.7 indicate suitability for factor analysis, with ≥0.8 being very suitable. The KMO values of the scales are 0.835, 0.774, 0.700, 0.850, and 0.855. These results show good structural validity of measurement tools and confirm that factor analysis is appropriate for all dimensions and the overall scale.

**Table 5:** Validity test results.

Dimensions	KMO	*P*	Number of items
*WorkMot*	0.835	<0.001	4
*IntCareBel*	0.774	<0.001	4
*ExtCareBel*	0.700	<0.001	3
*WorkDisf*	0.850	<0.001	4
*Overall scale*	0.855	<0.001	15

### 5.2 Descriptive statistics

The means, standard deviations, minima, medians, and maxima of all selected variables are presented in Table 6. The mean of *WorkMot* is 5.32, a relatively high level within the 0–7 range, indicating generally strong organizational commitment among employees, including emotional identification with the organization and willingness to stay long-term. The standard deviation is 1.40, showing some variation across individuals. The mean value of *Age* is 33.90, with a median of 32, implying that most respondents fall into early adulthood (ages 30–39). Similarly, most employees are the Han ethnicity, the proportion of women is slightly higher, and more than half are married. Variables related to work income and benefits such as *MonNetInc, AnnBonus20, Welfare*, as well as psychological and behavioral indicators such as *PromoteFut* and *IntCareBel*, also show distinct distributional characteristics. Overall, the distribution of variables reflects diversity in demographic traits, career development, and psychological states, providing a basis for analyzing the mechanisms influencing workplace proactivity.

**Table 6:** General description.

VarName	Obs	Mean	SD	Min	Median	Max
*WorkMot*	3,287	5.32	1.40	0	5.50	7
*Age*	3,287	33.90	9.78	16	32	72
*Gender*	3,287	0.45	0.50	0	0	1
*National*	3,287	0.03	0.16	0	0	1
*Marry*	3,287	0.57	0.50	0	1	1
*CurrTenure*	3,287	6.36	6.55	1	3.92	49.60
*CoTraining*	3,287	0.58	0.49	0	1	1
*MonNetInc*	3,287	7.21	3.56	0	8.52	15.60
*AnnBonus20*	3,287	4.22	4.22	0	5.30	12.80
*Welfare*	3,287	1.92	1.50	0	2	9
*OTDaysPWLM*	3,287	0.97	1.68	0	0	7
*WorkFormPref*	3,287	2.38	0.67	1	2	3
*Promote3yr*	3,287	0.22	0.41	0	0	1
*PromoteFut*	3,287	2.85	1.10	1	3	5
*LifeSat*	3,287	3.53	0.84	1	4	5
*RelatSat*	3,287	3.79	0.97	1	4	5
*WorkPresSat*	3,287	3.36	1.00	1	3	5
*IntCareBel*	3,287	3.81	0.69	1	4	5
*ExtCareBel*	3,287	2.70	0.89	1	2.67	5
*EAnnuity*	3,287	0.27	0.44	0	0	1
*SignContract*	3,287	0.83	0.37	0	1	1
*LabProtect*	3,287	3.92	0.81	1	4	5

### 5.3 Multicollinearity analysis

Table 7 reports the variance inflation factor (VIF) results. The results indicate that there is no significant multicollinearity among independent and control variables. The model specification is sound, and the estimation results are stable.

**Table 7:** Variance inflation factor.

Variable	VIF	1/VIF
*Age*	2.016	0.496
*MonNetInc*	1.674	0.597
*CurrTenure*	1.629	0.614
*Marry*	1.613	0.620
*AnnBonus20*	1.553	0.644
*RelatSat*	1.322	0.756
*PromoteFut*	1.292	0.774
*WorkPresSat*	1.244	0.804
*Welfare*	1.239	0.807
*CoTraining*	1.236	0.809
*LabProtect*	1.211	0.825
*Promote3yr*	1.186	0.843
*LifeSat*	1.166	0.858
*IntCareBel*	1.166	0.858
*SignContract*	1.096	0.912
*EAnnuity*	1.089	0.918
*WorkFormPref*	1.074	0.931
*ExtCareBel*	1.065	0.939
*Gender*	1.037	0.964
*OTDaysPWLM*	1.037	0.964
*National*	1.017	0.983
Mean VIF	1.284	

All VIF values are less than 5, indicating the absence of severe multicollinearity.

### 5.4 Baseline regression results

This study employs an OLS linear regression model for empirical analysis. Among the research variables, indicators such as *PromoteFut*, *LifeSat*, *RelatSat*, and *WorkPresSat*—measured using Likert scales—are all treated as continuous variables and incorporated into the regression model. This approach is sufficiently justified for the following reasons: First, the aforementioned indicators are composite indices formed by summing Likert-type equidistant items. These items exhibit good internal consistency, and the resulting summed scores display continuous gradient characteristics with sufficient variance, satisfying the basic statistical properties of continuous variables. This treatment enables the variables to effectively reflect gradations in the underlying constructs. Second, in empirical research within management, sociology, and other social sciences, treating summed Likert-scale indices as continuous variables in OLS linear regression models has become a well-established and mature research practice. This approach fully exploits the continuous information embedded in the data, more precisely captures the linear associations among variables, enhances the explanatory power of the model, and improves the precision of the results—aligning closely with the core analytical objective of this study to quantitatively examine the relationships between variables.

Table 8 column (1) reports the baseline regression results. The findings show that *Age, National, Marry, CurrTenure, CoTraining, Welfare, WorkFormPref, PromoteFut, LifeSat, RelatSat, WorkPresSat, IntCareBel,* and *LabProtect* have significant positive effects on employee organizational commitment, while *OTDaysPWLM* and *ExtCareBel* have significant negative effects. Column (2) presents the relative contribution rate of each variable, calculated using standardized regression coefficients to compare the contributions of the independent variables. The standardized regression coefficient indicates the change in the dependent variable (in standard deviation units) resulting from a one-standard-deviation change in the independent variable (with other variables held constant). The calculation formula is:


$$\text{Relative contribution rate}=\frac{|\beta_{i}|}{\Sigma |\beta_{j}|}$$

**Table 8:** Baseline regression results.

Variable	(1)	(2)
	*WorkMot*	Relative contribution rate
*Age*	0.012***	6.00%
	(3.81)	
*Gender*	−0.035	0.92%
	(−0.81)	
*National*	0.405***	3.42%
	(3.34)	
*Marry*	0.215***	5.64%
	(4.03)	
*CurrTenure*	0.012***	4.31%
	(3.01)	
*CoTraining*	0.165***	4.33%
	(3.32)	
*MonNetInc*	−0.013	2.39%
	(−1.61)	
*AnnBonus20*	0.003	0.60%
	(0.42)	
*Welfare*	0.044***	3.49%
	(2.93)	
*OTDaysPWLM*	−0.023*	2.01%
	(−1.72)	
*WorkFormPref*	0.068*	2.41%
	(1.91)	
*Promote3yr*	0.055	1.21%
	(1.11)	
*PromoteFut*	0.187***	10.90%
	(8.27)	
*LifeSat*	0.083***	3.69%
	(2.69)	
*RelatSat*	0.182***	9.33%
	(6.42)	
*WorkPresSat*	0.119***	6.33%
	(4.70)	
*IntCareBel*	0.355***	12.89%
	(9.29)	
*ExtCareBel*	−0.064**	3.03%
	(−2.53)	
*EAnnuity*	0.078	1.84%
	(1.56)	
*SignContract*	0.073	1.45%
	(1.09)	
*LabProtect*	0.324***	13.82%
	(10.08)	
_cons	0.041	/
	(0.17)	
*N*	3,287	
*R*^2^	0.271	

*t* statistics in parentheses, *p < 0.1, **p < 0.05, ***p < 0.01.

Age (*Age*), job tenure (*CurrTenure*), and participation in corporate trainings (*CoTraining*) collectively constitute the foundational ability resources of employees, and all three significantly enhance organizational commitment. Hypothesis 1 is thus supported.

The coefficient for *Age* is 0.012 (*p* < 0.01), with a relative contribution rate of 6.00%. As age increases and career stages progress, individuals accumulate work experience, problem-solving capabilities, and occupational cognition, enabling them to complete tasks more efficiently, reduce frustration, and gradually strengthen professional value identification, thereby elevating workplace proactivity (Kanfer and Ackerman, 2004). This finding supports the capability accumulation effect associated with age. It aligns with the core logic of COR, whereby individuals accumulate critical resources over time to enhance their capacity to meet work demands. The coefficient for *CurrTenure* is 0.012 (*p* < 0.01), with a contribution rate of 4.31%. Longer job tenure facilitates employees’ familiarity with organizational systems, mastery of job-specific skills, and embedding within internal networks, thereby reducing work-related friction and increasing behavioral autonomy. However, the modest contribution rate indicates that the marginal effect of tenure is relatively mild, suggesting that its enhancement of workplace proactivity is gradual and exhibits convergence, consistent with the law of diminishing marginal returns in resource accumulation. The coefficient for *CoTraining* is 0.165 (*p* < 0.01), with a contribution rate of 4.33%. Corporate training, as a structured resource provided by the organization, directly enhances employees’ job competence and sense of control through systematic empowerment, thereby increasing their willingness to engage proactively. Analyzed through the JD-R, this demonstrates that training, as a job resource, can buffer work pressure and stimulate workplace proactivity (Sesen and Ertan, 2022). Although training exerts a direct effect on ability enhancement, its overall contribution remains lower than that of core variables in the motivation and opportunity dimensions.

Overall, the ability dimension forms an important foundation for workplace proactivity, but it is not the most explanatory dominant dimension. This finding indicates that ability serves as a necessary but not sufficient condition for workplace proactivity; merely improving skills and experience is insufficient to maximally activate employees’ proactive engagement.

The motivation dimension exhibits the highest relative contribution rate and the clearest structural pattern among all variables, serving as the dominant source of workplace proactivity–its effect far exceeds the balanced perspective traditionally emphasized in prior research.

The coefficient for marital status (*Marry*) is 0.215 (*p* < 0.01), with a contribution rate of 4.51%. Married employees face stronger family responsibilities and demands for stability, making it easier to convert external pressures into intrinsic workplace proactivity and thereby demonstrating higher occupational engagement. This finding corroborates the driving role of “resource acquisition motivation” on behavior as posited in COR. The coefficient for non-monetary benefits (*Welfare*) is 0.044 (*p* < 0.01), with a contribution rate of 3.89%. The organizational care conveyed through benefits satisfies needs for security, respect, and belonging, facilitating the transformation of extrinsic incentives into intrinsic motivation. This aligns with SDT’ s emphasis on the three basic psychological needs - autonomy, competence, and relatedness—and is consistent with the formation mechanism of reciprocity norms in Social Exchange Theory. The coefficient for perceived opportunities for future promotion (*PromoteFut*) is 0.187 (*p* < 0.01), with a notably high contribution rate of 10.90%. Clear promotion pathways provide employees with goal-directed and long-term incentives, significantly strengthening self-efficacy and sustained engagement intention, thus constituting a key driver within the motivation dimension. Promotion expectations simultaneously fulfill individuals’ needs for growth and competence while reinforcing long-term social exchange relationships between employees and the organization. The coefficients for life satisfaction (*LifeSat*), interpersonal relationship satisfaction (*RelatSat*), and work pressure satisfaction (*WorkPresSat*) are 0.083, 0.182, and 0.119, respectively, all significant at the 1% level, with contribution rates of 4.58, 9.33, and 6.24%. These three factors stably enhance workplace proactivity through distinct pathways: life foundation, social support, and pressure fit, among which interpersonal relationship satisfaction makes a particularly prominent contribution. This highlights that high-quality interpersonal relationships serve as a critical carrier of social exchange, a key emotional resource emphasized in COR, and simultaneously satisfy the relatedness need in SDT, underscoring the irreplaceable role of emotional motivation and social support in contemporary workplaces. More importantly, the coefficient for internal career locus of control (*IntCareBel*) is 0.355 (*p* < 0.1), achieving the highest contribution rate of 12.89%. Individuals holding an internal locus of control attribute career outcomes to their own efforts, forming a stable motivational system characterized by clear goals, self-reinforcement, and internalized responsibility. Drawing on SDT, when individuals perceive behavior as driven by autonomous volition and outcomes as controllable by themselves, intrinsic motivation reaches its peak. In contrast, the coefficient for external career locus of control (*ExtCareBel*) is −0.064 (*p* < 0.05), with a contribution rate of 3.03%, significantly undermining workplace proactivity.

Thus, Hypothesis 2 is supported. Moreover, the above results demonstrate that the motivation dimension is not merely a simple aggregation of disparate factors, but a structured system centered on internal career locus of control, with promotion expectations and interpersonal support as key pillars. This conclusion transcends the traditional balanced assumption of AMO theory and clearly establishes the dominant role and hierarchical structure of the motivation dimension.

The opportunity dimension provides external conditions for the enactment of ability and the release of motivation. Its contribution rate ranks second only to the motivation dimension and exhibits distinct structural characteristics.

The coefficient for ethnicity (*National*) is 0.405 (*p* < 0.01), with a contribution rate of 3.42%. Under the combined influence of cultural respect, institutional support, and identity affirmation, ethnic minority employees are more inclined to demonstrate higher workplace proactivity in order to gain organizational recognition through positive performance. This result illustrates the empowering role of an inclusive environment as a critical work resource in fostering proactivity. The coefficient for work arrangement preference (*WorkFormPref*) is 0.068 (*p* < 0.1), with a contribution rate of 2.41%. The alignment between individual preferences and actual work settings reduces communication costs and enhances focus, thereby stably promoting workplace proactivity (Dullah et al., 2023). Drawing on SDT, when the work environment matches individual needs, intrinsic motivation is more readily activated. The coefficient for perceived labor protection (*LabProtect*) is 0.324 (*p* < 0.01), with a strikingly high contribution rate of 13.82%. The sense of occupational security provided by labor protection significantly reduces employees’ risk concerns, enabling them to invest more boldly and assume greater responsibility. From the perspective of the JD-R, labor protection constitutes a classic protective work resource; from COR, it prevents potential resource loss; from Social Exchange Theory, it strengthens employees’ trust in and reciprocal commitment to the organization. This finding elevates the opportunity dimension from a merely “supportive condition” to a foundational and prerequisite driver, substantially enriching the conceptual scope of “opportunity” within the AMO framework.

The coefficient for average weekly overtime (*OTDaysPWLM*) is −0.023 (*p* < 0.1), with a contribution rate of 2.01%. Excessive overtime represents a prototypical high job demand; prolonged overtime continuously depletes individuals’ physiological and psychological resources, violating the fundamental principle of COR that “individuals strive to maintain and protect existing resources.” This leads to energy depletion and psychological resistance, directly eroding the foundation of workplace proactivity. Thus, Hypothesis 3 is supported.

In summary, the core function of the opportunity dimension lies in providing psychological safety and boundary protection, rather than serving merely as external support. The extreme importance of perceived labor protection highlights that, in contemporary workplaces, a sense of security constitutes the baseline condition for activating proactivity.

### 5.5 Robustness check

To test whether the core theoretical relationships are limited by a single-polar measurement approach, this study conducts a robustness check by replacing the dependent variable. The results are presented in Column (1) of Table 9. The questionnaire includes items related to turnover intention, specifically as follows: (1) “I often think about quitting my job”; (2) “I am always looking for a better job”; (3) “I may look for another job next year”; (4) “There is little to gain from staying in this job.” These items are equally weighted (1:1:1:1) and then summed to construct a composite measure of employees’ turnover intention (*WorkDisf*), which is used as a substitute for the dependent variable.

**Table 9:** Robustness check.

Variable	(1)	(2)
	*WorkDisf*	*WorkMot*
*Age*	−0.022***	0.012***
	(−4.79)	(3.25)
*Gender*	0.028	−0.035
	(0.45)	(−0.76)
*National*	−0.367*	0.405***
	(−1.92)	(2.92)
*Marry*	−0.260***	0.215***
	(−3.31)	(3.89)
*CurrTenure*	−0.010	0.012**
	(−1.62)	(2.52)
*CoTraining*	−0.235***	0.165***
	(−3.34)	(3.20)
*MonNetInc*	0.043***	−0.013
	(3.88)	(−1.53)
*AnnBonus20*	0.007	0.003
	(0.81)	(0.40)
*Welfare*	−0.046**	0.044***
	(−2.16)	(2.76)
*OTDaysPWLM*	0.039**	−0.023
	(2.10)	(−1.59)
*WorkFormPref*	−0.074	0.068*
	(−1.48)	(1.83)
*Promote3yr*	−0.190**	0.055
	(−2.46)	(1.08)
*PromoteFut*	−0.217***	0.187***
	(−6.82)	(7.62)
*LifeSat*	−0.210***	0.083***
	(−4.77)	(2.61)
*RelatSat*	−0.088**	0.182***
	(−2.31)	(6.05)
*WorkPresSat*	0.016	0.119***
	(0.45)	(4.43)
*IntCareBel*	−0.265***	0.355***
	(−5.04)	(8.79)
*ExtCareBel*	0.341***	−0.064**
	(8.93)	(−2.31)
*EAnnuity*	0.198***	0.078
	(2.63)	(1.47)
*SignContract*	−0.073	0.073
	(−0.78)	(0.96)
*LabProtect*	−0.373***	0.324***
	(−8.68)	(9.05)
_cons	6.361***	0.041
	(18.57)	(0.16)
*N*	3,287	3,287
*R*^2^	0.186	0.271

(1) WorkDisf represents the total score for turnover intention, calculated as the equally weighted sum of four reverse-coded items (0–7 scale). Higher scores indicate stronger turnover intention. (2) *t* statistics in parentheses, *p < 0.1, **p < 0.05, ***p < 0.01.

This study selected turnover intention as the robustness check variable for organizational commitment. The rationale for this choice stems from the mature understanding of organizational behavior regarding job burnout and its consequences. According to meta-analytic evidence from the perspective of COR (Lee and Ashforth, 1996), turnover intention represents a direct behavioral intention outcome of job burnout, indicating that turnover intention is not an isolated attitude but rather a clear behavioral withdrawal tendency directed by a series of negative psychological states. Therefore, theoretically, organizational commitment (positive attitude) and turnover intention (negative behavioral tendency) constitute the two opposing yet logically isomorphic endpoints on the continuous spectrum of employees’ attitudes toward the organization. Examining whether the direction of influence of the independent variables on turnover intention is opposite to their effect on organizational commitment essentially constitutes a structural cross-validation of the core influencing mechanisms from the perspective of preventing negative behavioral outcomes. Meanwhile, the two constructs are structurally symmetric, both measured using four items on a 0–7 Likert scale, ensuring comparable statistical properties and avoiding bias arising from differences in measurement scales.

Results in Column (1) show that most variables remain robust across the two models, with the direction of effects exhibiting strict mirror symmetry.

Among these variables, *WorkPresSat* is significant only in the *WorkMot* model. A plausible explanation is that a moderate level of perceived pressure directly enhances workplace proactivity, but its ability to reduce disengagement is limited. Even when perceived pressure is well-adjusted, employees may still experience job dissatisfaction due to other factors. Thus, *WorkPresSat* remains significant exclusively in *WorkMot.*

In contrast, *Promote3yr, MonNetInc*, and *EAnnuity* are significant only in the *WorkDisf* model. First, past promotions can mitigate employees’ feelings of unfairness and thus lower disengagement, but may not significantly enhance motivation since the achievement of promotion goals could weaken subsequent motivational drive. This explains why *Promote3yr* is significant only for *WorkDisf*. Second, for *MonNetInc*, high-income employees in the sample may also face heavier workloads; increased income may therefore intensify negative work evaluations. However, in the *WorkMot* model, this effect may be offset by the sense of security associated with higher pay, resulting in significance only for *WorkDisf*. Third, *EAnnuity*, as a long-term benefit provided by organizations to employees, has delayed incentive effects and may not immediately enhance short-term workplace proactivity. Nevertheless, the risk of losing such benefits due to potential job departure might increase dissatisfaction regarding career flexibility, making it significant only in the *WorkDisf* model.

The replacement of the dependent variable confirms the stability of the core mechanisms identified in this study. The main results remain unaffected by potential biases arising from monopolar measurement approaches. Meanwhile, the differential significance of certain variables reveals an inherent asymmetry between the drivers of positive workplace attitudes and the factors that mitigate negative ones. This provides more refined boundary conditions and contextualized evidence for the application of the AMO framework across the dual dimensions of employee attitudes. This further suggests that enhancing workplace proactivity and reducing workplace disengagement require differentiated strategies: the former should emphasize strengthening intrinsic motivation and environmental support, while the latter should focus on alleviating external constraints and perceptions of unfairness. Only through the combined use of these approaches can organizations comprehensively improve employees’ workplace attitudes.

Furthermore, the main regression was also estimated using both firm-level clustered robust standard errors and bootstrap resampling methods, with results presented in Column (2). These adjustments correct for within-group correlation of error terms among employees from the same company and mitigate potential biases arising from non-normal residuals and small-sample issues. The results show that the signs, statistical significance, and magnitudes of the independent variables remain basically consistent with those in the main model, with no substantial changes attributable to the alternative standard error specifications.

### 5.6 Heterogeneity analysis

Table 10 presents the heterogeneity analysis across four age groups. The results reveal substantial differences in the determinants of workplace proactivity at different career stages. To deepen the understanding of the driving mechanisms of workplace proactivity, this study further explores its heterogeneity across different age groups from the perspectives of the life course perspective and career stage theory. Super (1953) proposed a career development theory that divides individuals’ career lifespan into four stages—exploration, establishment, maintenance, and disengagement—each characterized by distinct core tasks, thereby providing a theoretical framework for understanding age-related differences in workplace proactivity. Meanwhile, Sustainable Human Resource Management (SHRM) emphasizes that organizational support should be dynamically aligned with employees’ lifecycle needs to achieve a win-win outcome for long-term career development and organizational effectiveness (Jung and Takeuchi, 2018). This study divides the sample into four age groups—under 29 years (youth), 30–39 years (early adulthood), 40–54 years (mid-adulthood), and 55 years and above (late adulthood) – corresponding, respectively, to the exploration, establishment, maintenance, and disengagement stages. This approach aims to reveal the stage-specific characteristics of workplace proactivity and its antecedents, thereby providing empirical evidence for stage-tailored interventions in SHRM practice (Table 10).

**Table 10:** Heterogeneity test.

Variable	Age under 29 (Youth)	Age 30–39 (Early adulthood)	Age 40–54 (Mid-adulthood)	Age above 55 (Late adulthood)
	*WorkMot*	*WorkMot*	*WorkMot*	*WorkMot*
*Gender*	0.058	−0.171**	0.034	−0.241
	(0.83)	(−2.21)	(0.38)	(−1.06)
				[−0.690, 0.209]
*National*	0.441**	0.294*	0.281	0.756
	(2.16)	(1.65)	(1.01)	(1.40)
				[−0.314, 1.825]
*Marry*	0.370***	0.196**	0.075	−0.504**
	(4.35)	(2.21)	(0.59)	(−2.23)
				[−0.954, −0.054]
*CurrTenure*	0.014	0.020**	0.010	0.011
	(1.25)	(2.53)	(1.52)	(1.09)
				[−0.009, 0.032]
*CoTraining*	0.106	0.193**	0.161	0.085
	(1.34)	(2.29)	(1.51)	(0.27)
				[−0.527, 0.696]
*MonNetInc*	−0.012	−0.012	0.003	−0.025
	(−1.05)	(−0.73)	(0.13)	(−0.49)
				[−0.125, 0.076]
*AnnBonus20*	−0.001	0.005	0.004	−0.018
	(−0.09)	(0.43)	(0.30)	(−0.61)
				[−0.077, 0.041]
*Welfare*	0.029	0.063***	0.023	0.064
	(1.11)	(2.69)	(0.64)	(1.18)
				[−0.044, 0.172]
*OTDaysPWLM*	−0.018	−0.022	−0.027	−0.012
	(−0.82)	(−1.01)	(−0.97)	(−0.19)
				[−0.139, 0.115]
*WorkFormPref*	0.005	0.147**	0.075	0.241
	(0.10)	(2.33)	(0.97)	(1.05)
				[−0.214, 0.697]
*Promote3yr*	0.026	0.142*	0.035	−0.217
	(0.31)	(1.77)	(0.31)	(−0.67)
				[−0.863, 0.430]
*PromoteFut*	0.216***	0.185***	0.162***	0.135
	(5.86)	(4.46)	(3.66)	(1.33)
				[−0.067, 0.336]
*LifeSat*	0.113**	0.046	0.114*	−0.039
	(2.11)	(0.87)	(1.81)	(−0.38)
				[−0.244, 0.165]
*RelatSat*	0.161***	0.145***	0.240***	0.241*
	(3.31)	(2.96)	(4.56)	(1.76)
				[−0.030, 0.512]
*WorkPresSat*	0.175***	0.147***	0.014	0.031
	(4.58)	(2.93)	(0.28)	(0.35)
				[−0.145, 0.207]
*IntCareBel*	0.301***	0.395***	0.328***	0.614***
	(4.51)	(6.23)	(4.24)	(3.85)
				[−0.298, 0.931]
*ExtCareBel*	−0.093**	0.009	−0.126**	−0.058
	(−2.08)	(0.22)	(−2.43)	(−0.50)
				[−0.288, 0.171]
*EAnnuity*	0.036	0.099	0.071	−0.260
	(0.41)	(1.21)	(0.67)	(−0.99)
				[−0.778, 0.259]
*SignContract*	0.076	−0.054	0.240	0.274
	(0.75)	(−0.42)	(1.63)	(1.14)
				[−0.204, 0.752]
*LabProtect*	0.376***	0.286***	0.313***	0.259*
	(6.75)	(5.60)	(4.67)	(1.70)
				[−0.044, 0.561]
_cons	0.208	0.258	0.866	0.990
	(0.51)	(0.62)	(1.62)	(1.18)
*N*	1,264	1,143	762	118
*R*^2^	0.286	0.258	0.241	0.391

(1) Values in square brackets represent 95% confidence intervals. (2) *t* statistics in parentheses, *p < 0.1, **p < 0.05, ***p < 0.01.

*Gender* exhibits a significant negative effect only in the 30–39 age group (early adulthood) (*β* = −0.171, *p* < 0.05), with no significant differences observed in the other stages. Drawing on career stage theory, during the establishment stage, female employees simultaneously confront the dual tasks of establishing professional identity and deepening family roles. Traditional gender role expectations substantially constrain their willingness to invest in their careers, resulting in significantly lower workplace proactivity compared to males. In the youth stage, individuals have not yet become deeply involved in family roles, rendering gender differences negligible. In the mid- and late-adulthood stages, conflicts between family responsibilities and career development are markedly alleviated, and the influence of gender on workplace proactivity dissipates accordingly. The data indicate that the effect of gender on workplace proactivity is not constant but is concentrated in the critical conflict period of career development.

*Marry* (marital status) exhibits pronounced stage-specific heterogeneity. In the under 29 (youth) and 30–39 (early adulthood) groups, marriage acts as a significant positive driver, with coefficients of 0.370 and 0.196, respectively. In contrast, it shows a significant negative effect in the 55 years and above (late adulthood) group, with a coefficient of −0.504. Drawing on career stage theory, the youth and early adulthood stages represent the critical period during which individuals simultaneously establish professional identity and family roles. Married employees, burdened by greater family responsibilities, place a higher value on career stability and financial security, resulting in elevated workplace proactivity. By late adulthood, however, the primary task shifts from career advancement to retirement transition, with reduced family obligations and diminished occupational goals, thereby reversing the motivational effect of marriage on career investment. This pattern aligns closely with the lifecycle support principle of SHRM, suggesting that organizations should implement differentiated support strategies tailored to employees’ core needs at each stage: strengthening career stability assurances during youth and early adulthood, while providing retirement transition support in late adulthood to better match the demands of different life and career phases.

The regression results show that *CurrTenure* (*β* = 0.020), *CoTraining* (*β* = 0.193), and *Welfare* (*β* = 0.063) exhibit significant positive effects only in the 30–39 age group (early adulthood). Early adulthood represents the critical period for accumulating professional capabilities and establishing positional influence. Job tenure directly affects promotion eligibility, skill training precisely addresses the need to overcome career bottlenecks, and non-monetary benefits effectively alleviate dual pressures from family and career demands. From the perspective of the AMO framework, these three variables correspond, respectively, to Ability, Motivation, and Opportunity: tenure accumulation enhances ability, training strengthens motivation, and benefits create opportunities. In other age stages, the effects of these variables are not significant, indicating that their motivational impact is highly stage-specific—effective only during the establishment stage, when individuals’ demand for ability enhancement and resource support is most urgent, thereby translating into workplace proactivity. *WorkFormPref* (*β* = 0.147) and *Promote3yr* (*β* = 0.142) also show significant positive effects exclusively in the 30–39 age group (early adulthood). During the establishment stage, employees have a stronger need for work flexibility; arrangements that match their preferred work forms can effectively reduce role conflict and enhance workplace proactivity. At the same time, short-term promotion expectations serve as a core motivator driving career investment, and well-designed promotion pathways can strongly activate proactivity in this stage. In other stages, the incentive effects of work arrangement preference and short-term promotion expectations are relatively weakened, which is highly consistent with the shifting core tasks across career stages.

*LifeSat* shows significant positive effects in the under 29 (youth) group (*β* = 0.113, *p* < 0.05) and the 40–54 (mid-adulthood) group (*β* = 0.114, *p* < 0.1), but is not significant in the 30–39 (early adulthood) or 55 years and above (late adulthood) groups. A plausible explanation is that, during the youth stage, life satisfaction serves as a foundational factor for organizational integration and alleviation of adaptation stress; in mid-adulthood, it provides sustained support for stable engagement during the maintenance phase of the career. *WorkPresSat* exhibits significant positive effects in the under 29 (youth) group (*β* = 0.175, *p* < 0.01) and the 30–39 (early adulthood) group (*β* = 0.147, *p* < 0.01), but becomes non-significant in groups aged 40 and above. During the exploration and establishment stages, employees are more sensitive to perceptions of work pressure, and satisfaction with pressure management directly influences their willingness to invest in their careers. In contrast, during mid-to-late adulthood, career stability increases, attenuating the marginal impact of pressure satisfaction. These findings collectively indicate that the driving role of individual well-being on workplace proactivity is stage-specific.

*ExtCareBel* exhibits significant negative effects in the under 29 (youth) group (*β* = −0.093, *p* < 0.05) and the 40–54 (mid-adulthood) group (*β* = −0.126, *p* < 0.05), but is not significant in the 30–39 (early adulthood) or 55 years and above (late adulthood) groups. During the exploration and maintenance stages, an excessive belief in external care undermines individuals’ occupational autonomy, thereby significantly reducing workplace proactivity. In contrast, its influence remains relatively negligible during early adulthood and late adulthood. This finding suggests that organizations should place particular emphasis on fostering employees’ self-efficacy, especially during the exploration and maintenance stages, to enhance their sense of career autonomy and thereby elevate workplace proactivity.

By embedding workplace proactivity within the dynamic perspective of the life course and career stages, this study reveals the stage-specific nature of various antecedent variables. The significant effects of *Gender*, *CurrTenure*, *CoTraining*, *Welfare*, *WorkFormPref*, and *Promote3yr* are concentrated in the 30–39 age group—the establishment stage—aligning closely with the core characteristics of this phase: establishment of professional identity, capability advancement, and role conflict. In contrast, the influences of life satisfaction, work pressure satisfaction, and external career locus of control are more pronounced during the exploration and maintenance stages. These findings deepen the understanding of the driving mechanisms of workplace proactivity and highlight the need for organizations to dynamically adjust human resource strategies in accordance with employees’ current career stages, thereby achieving a sustainable win-win outcome between individual career development and long-term organizational effectiveness.

## 6 Conclusion and policy implications

### 6.1 Research conclusion

Using the 2021 wave of the CGSS as the empirical sample, this study investigates the determinants of employee workplace proactivity within the AMO framework. The empirical results indicate that among ability-related variables, *Age* (0.012***), *CurrTenure* (0.012***), and *CoTraining* (0.165***) exert significant positive effects on workplace proactivity. At the motivation level, *Marry* (0.215***), *Welfare* (0.044***), *PromoteFut* (0.187***), *LifeSat* (0.083*), *RelatSat* (0.182***), *WorkPresSat* (0.119***), and *IntCareBel* (0.355***) positively influence work proactivity, whereas *ExtCareBel* (−0.064**) shows a significant negative effect. At the opportunity level, *National* (0.405***), *WorkFormPref* (0.068*), and *LabProtect* (0.324***) demonstrate significant positive associations with workplace proactivity, while *OTDaysPWLM* (−0.023*) negatively affects workplace proactivity. By calculating the relative contribution rates, *LabProtect* and *IntCareBel* emerge as the two core drivers of workplace proactivity, with contribution rates significantly higher than those of other variables. Overall, the explanatory power of the motivation dimension and the opportunity dimension substantially exceeds that of the ability dimension, challenging the traditional assumption of balanced contributions across the three dimensions within the AMO framework. The heterogeneity analysis further reveals that early adulthood (30–39 years old) is the stage in which the influencing factors of workplace proactivity are most concentrated. Additionally, interpersonal relationship satisfaction, internal career locus of control, and perceived labor protection serve as consistent and stable drivers across all age groups.

### 6.2 Policy implications

#### 6.2.1 Ability dimension

“Ability” is conceptualized as employees’ occupational skills, accumulated experience, and capacity for knowledge updating. The core intervention logic lies in deploying stage-specific resource investments to match the ability development needs across different career stages, thereby progressing from ability enhancement to increased sense of workplace control, ultimately activating employee workplace proactivity.

At the organizational level, the first consideration is to establish a stage-differentiated ability development system. The empirical results show that age, job tenure, and active participation in corporate training exert positive effects on workplace proactivity, with heterogeneous effects across different age groups. Accordingly, corporates may consider establishing an age-segmented capability development system and designing differentiated skills-training and job-training programs to help employees accumulate work experience and improve job competence. Meanwhile, corporates should refine their internal training mechanisms by expanding the coverage of professional training programs, enriching training contents and formats, and encouraging employees to engage in skill-enhancement and career-development activities, thereby increasing their work engagement and proactivity through capability improvement. At the national level, it is necessary to improve the support system for occupational education and lifelong learning. The national government should introduce incentive policies for school-corporate cooperation, granting tax reductions and subsidy support to corporates participating in youth skill development, while standardizing the implementation criteria for the dual occupational training model. At the same time, a lifelong occupational skills training subsidy system should be established, with special training funds set up to address the skill-updating needs of middle-aged and older employees. This would reduce the training costs borne by corporates and provide institutional safeguards for ability-building efforts at the organizational level.

#### 6.2.2 Motivation dimension

Motivation is primarily manifested through employees’ internal beliefs, emotional needs, and perceptions of external incentives. The core intervention logic involves optimizing organizational incentives and strengthening psychological support, drawing on Social Exchange Theory and SDT, to facilitate the internalization of extrinsic incentives into intrinsic beliefs, ultimately achieving sustained workplace proactivity.

At the organizational level, corporates should optimize differentiated incentive and psychological support systems. Regarding differentiated incentives based on marital status and age, organizations can target young and early-adulthood married employees by offering flexible working hours, childcare subsidies, paid family leave, and similar supports to amplify the positive effect of marriage on workplace proactivity. For employees in late adulthood, corporates should design retirement transition incentives, such as flexible work positions and rewards for experience contribution, in order to mitigate the motivational decline associated with retirement expectations. In terms of welfare experience and psychological well-being, corporates should enrich customized non-monetary benefits, including free work meals, funding for interest-based clubs, generous paid leave policies, and other measures to safeguard work-life balance. Corporates should also establish a workplace well-being index monitoring mechanism, supported by dedicated psychological counseling hotlines, stress management training programs, and the creation of a positive and supportive office environment, thereby strengthening employees’ emotional attachment and sense of belonging. At the level of organizational culture shaping, corporates can conduct workshops on attribution training and self-efficacy enhancement, drawing on positive psychology practices to guide employees toward attributing career success to their own efforts and abilities. Additionally, through career mentoring sessions and success-story sharing events, organizations can weaken the negative influence of external locus of control beliefs and solidify the foundation of intrinsic motivation.

At the national level, it is essential to improve the policy system for family support and silver economy employment. The national government should implement preferential tax policies, granting special tax deductions to corporates that adopt family-friendly welfare measures. At the same time, public service provision in childcare and elderly care should be expanded to alleviate the family burden on employees. Drawing on Singapore’s Senior Employment Credit (SEC) and CPF Transition Offset (CTO) policies, employment subsidies for older workers and provident fund burden reduction mechanisms should be established (Kwan and Asher, 2022). These institutional measures will enhance the occupational motivation of employees in late adulthood from the policy level, thereby providing macro-level support for motivation activation efforts at the organizational level.

#### 6.2.3 Opportunity dimension

The opportunity is primarily manifested through employees’ developmental space, rights protection, and an inclusive environment. Its core intervention logic relies on the JD-R and COR, by optimizing the organizational environment and enforcing rigid safeguards for rights, thereby providing external support for the enactment of employees’ abilities and the release of their motivation.

At the organizational level, building an inclusive environment and a scientific management system is essential. Corporates are encouraged to systematically construct a highly inclusive organizational ecosystem by establishing mechanisms for ethnic cultural exchange, respecting the customs and needs of different ethnic groups, providing multilingual communication support, and ensuring equal development opportunities for employees of all ethnic backgrounds. Regarding work arrangements, based on employee preference surveys, corporates should optimize office facilities and collaboration tools for those who prefer on-site work, while establishing outcome-oriented performance evaluation mechanisms for those who prefer remote work, thereby minimizing the loss of proactivity caused by mismatches between preferred and actual work forms. In terms of work intensity management, corporates should implement working-hour monitoring and overtime approval mechanisms, reduce unnecessary overtime through process optimization and technological enablement, and strictly enforce overtime compensation policies. These measures prevent excessive resource depletion from eroding workplace proactivity.

At the national level, it is essential to strengthen the labor protection and rights supervision system. The national government should improve labor protection laws and regulations, refine protection standards tailored to different industries and job positions, and conduct regular enforcement inspections to compel corporates to implement core requirements such as workplace safety measures and provision of protective equipment. A labor protection satisfaction monitoring mechanism and a complaint handling system should be established to ensure smooth channels for employees to voice rights-related appeals. For emerging employment forms and flexible work arrangements, specialized labor protection regulations should be promulgated to achieve full coverage of rights protection, thereby providing rigid institutional constraints to support opportunity provision at the organizational level.

### 6.3 Limitations

This study employs data from the 2021 wave of the CGSS to examine the determinants of employee workplace proactivity. However, several limitations remain. First, there are inherent constraints related to data timeliness and cross-sectional design. Because the analysis relies on a single-year cross-sectional dataset (CGSS 2021), it is difficult to capture the long-term dynamic trends of factors influencing workplace proactivity. Second, there are potential limitations concerning sample representativeness. Although the CGSS is a comprehensive survey with broad coverage, the selection of employee samples may not fully account for the specificities of certain industries or employment arrangements. As a result, the applicability of the conclusions to segmented groups requires further validation. Furthermore, common method bias arising from self-reported data may affect the accuracy of the results, as participants’ subjective evaluations could introduce bias into the findings. Omitted variable bias is also a noteworthy concern. Additionally, endogeneity issues may be present—for instance, more proactive employees might be more inclined to participate in training, which in turn could influence their level of workplace proactivity. Future research could adopt a longitudinal design, utilizing tracking survey data to analyze temporal changes in workplace proactivity and its influencing factors. Studies should also explore interaction effects within the AMO framework, examining how the interplay among ability, motivation, and opportunity jointly affects workplace proactivity. Additionally, cross-national comparative studies would offer a broader and more generalizable perspective for understanding workplace proactivity across diverse cultural and economic contexts.

## Data Availability

Publicly available datasets were analyzed in this study. This data can be found here: all the data used in this paper are from the “Chinese General Social Survey (CGSS)” project, which is hosted by the China Data Center for Social Surveys at Renmin University of China, and can be obtained at http://www.cnsda.org/index.php?r=projects/view&id=65635422.

## Footnotes

1
https://www.cnsda.org/index.php?r=projects/view&id=92173417
